# Progress in Pediatrics

**Published:** 1900-11-24

**Authors:** 


### PROGRESS IN PEDIATRICS.

**Day Terrors (Pavor Diurnus).**—This affection is to be distinguished from night terrors in that the latter occur while the patient is asleep, and the former while he is awake. It is a rare condition, of which Henoch has recorded two cases, and G. F. Still[14] has recently published three more. The symptoms are fairly constant. A nervous, excitable child, in the midst of his ordinary occupations, will suddenly and without any apparent cause begin to scream. He looks terrified, rushes to his mother, says some person is coming after him, or that he hears something alarming, and cannot be easily pacified. There is no loss of consciousness. The attacks may vary in duration from a few seconds to a quarter of an hour, and in frequency from one in a fortnight up to twenty in a day. They may or may not be associated with night terrors. The fact that the two conditions are associated in some cases points to a similarity in causation, and disproves the common assumption that sleep is a necessary factor in the production of night terrors. Dr. Still discusses the relation of these terrors to epilepsy, and does not find that the subjects of them are specially liable to that affection. He concludes that pavor diurnus is a paroxysmal psycho-neurosis allied to, but not identical with, epilepsy on the one hand and hysteria and insanity on the other. In its treatment he has obtained the best results by curing the condition of chronic intestinal catarrh which is usually present as the result of bad feeding. For this purpose simple plain food and the use of citrate of potash with compound decoction of aloes will be found useful. Sedatives in the form of bromides and belladonna will be required for a time. The nervous character of the child must also be recognised, and both work and play regulated so as to avoid mental strain and excitement.

**Measles complicated with Meningitis and Spinal Myelitis.**—A very interesting case of this nature, terminating in recovery, is recorded by E. F. Eliot.[15] The patient was a girl of eighteen years, healthy up to the onset of measles. On the fourth day after the eruption epileptiform seizures occurred, and were followed later by signs of spinal irritation. There was incontinence of urine and fæces, the lower limbs were paralysed, the upper limbs were paretic, and the knee jerks and plantar reflexes were abolished. The condition for some time was apparently hopeless, but after a prolonged illness complete recovery eventually took place.

**Myotonia Congenita (Thomsen's Disease).**—A case of this rare affection was shown by Leonard Guthrie[16] at a meeting of the Medical Society of London. The patient was a boy ten years old, who had been brought to the hospital because of the awkwardness of his movements. Transient spasticity affected both the lower and the upper extremities. He took hold of anything with difficulty, and could not let go easily. Sometimes he had to open out the fingers of one hand with the other before being able to grasp an object. He had a difficulty in feeding himself at times. His gait when starting was jerky, and his progression up a stair was laboured. The condition of spasm was much more marked after rest, and was lessened after exercise or when his attention was distracted from himself. The spasm persisted during sleep. Although the symptoms pointed clearly to Thomsen's disease, there was no family history of this affection in this case.

[14] The Lancet, Feb. 3, 1900. [15] Ibid., Feb. 24, 1900. [16] Ibid., April 14, 1900.

